# Children Can Implicitly, but Not Voluntarily, Direct Attention in Time

**DOI:** 10.1371/journal.pone.0123625

**Published:** 2015-04-16

**Authors:** Katherine A. Johnson, Emma Burrowes, Jennifer T. Coull

**Affiliations:** 1 School of Psychological Sciences, University of Melbourne, Parkville, Australia; 2 Laboratoire des Neurosciences Cognitives, Aix-Marseille Université and CNRS, 3 Place Victor-Hugo, Marseille, France; University of Groningen, NETHERLANDS

## Abstract

Children are able to use spatial cues to orient their attention to discrete locations in space from around 4 years of age. In contrast, no research has yet investigated the ability of children to use informative cues to voluntarily predict when an event will occur in time. The spatial and temporal attention task was used to determine whether children were able to voluntarily orient their attention in time, as well as in space: symbolic spatial and temporal cues predicted where or when an imperative target would appear. Thirty typically developing children (average age 11 yrs) and 32 adults (average age 27 yrs) took part. Confirming previous findings, adults made use of both spatial and temporal cues to optimise behaviour, and were significantly slower to respond to invalidly cued targets in either space or time. Children were also significantly slowed by invalid spatial cues, demonstrating their use of spatial cues to guide expectations. In contrast, children’s responses were not slowed by invalid temporal cues, suggesting that they were not using the temporal cue to voluntarily orient attention through time. Children, as well as adults, did however demonstrate signs of more implicit forms of temporal expectation: RTs were faster for long versus short cue-target intervals (the variable foreperiod effect) and slower when the preceding trial’s cue-target interval was longer than that on the current trial (sequential effects). Overall, our results suggest that although children implicitly made use of the temporally predictive information carried by the length of the current and previous trial’s cue-target interval, they could not deliberately use symbolic temporal cues to speed responses. The developmental trajectory of the ability to voluntarily use symbolic temporal cues is therefore delayed, relative both to the use of symbolic (arrow) spatial cues, and to the use of implicit temporal information.

## Introduction

Anticipating *where* in space and *when* in time an event will occur are basic survival skills. Knowing where or when an event will occur allows attentional resources to be directed towards a discrete location in space, or a moment in time, so as to enhance sensorimotor processing of stimuli occurring at that point. Directing or “orienting” of attention in space or through time allows us to optimize our behavior by avoiding accidents, taking advantage of opportunities, and preserving energy until action is needed [1, 2]. Most research on attentional orienting has focused on directing attention in space [3] and has been conducted mainly with adult participants. Significantly less research has focused on the ability to voluntarily orient attention in time [4] and even less on the developmental nature of temporal attention in children [5].

To direct attention to one location rather than another, one must first be able to perceive space. Infants have quite refined spatial perceptual skills [6] and readily orient their spatial attention to different stimuli in the environment [7]. The exogenous orienting system directs attention automatically to salient stimuli, whilst the endogenous system is activated voluntarily in response to cues that stipulate the location of an upcoming target [8]. Exogenous covert attention studies in infants aged from three months suggest that even at this young age, children are able to shift their attention spatially [9, 10]. Moreover, reliable, valid tasks, developed to test endogenous spatial shifting of attention in adults [11, 12], have shown that school-aged children are also able to voluntarily direct attention to different locations in space [13, 14].

In contrast with spatial orienting of attention, the developmental aspects of orienting attention through time are unclear. In order to direct one’s attention to one time point rather than another, one must first be able to perceive time. There are different forms of timing, including (1) estimating the duration of an event, (2) determining if an event occurred before or after another event, also known as temporal order judgment, and (3) predicting when an event will occur based on regularities in the environment or temporal cues. Research into timing in children has focused primarily on the first two forms. For example, infants can detect temporal deviants in rhythmic auditory sequences [15–17] and show evidence of scalar timing during duration discrimination [18–20]. By 3 years of age children show the same fundamental properties of time perception as adults, even though they are less accurate in making duration judgments [21, 22]. By 7 years, children are able to determine the correct temporal order of two acoustic events [23]. The ability to estimate time precisely improves during childhood [22, 24] and may be associated with the development of working memory, sustained attention, cognitive flexibility [25] and information processing speed [24], most likely underpinned by brain maturation processes [26]. Developmental research into the third form of timing, predicting when an event will occur in order to optimize behavior, is sparser [5, 27] and has often focused on findings that music and rhythm implicitly help children acquire and develop language, reading and attentional control [28–31]. Moreover, to our knowledge, no research has yet investigated the ability of children to use informative cues to voluntarily predict when an event will occur.

In adults, the behavioural and neural profiles of spatial and temporal attentional orienting have been compared and contrasted in functional neuroimaging [32], electrophysiological [33] and psychopharmacological [34, 35] investigations. In this paradigm, symbolic (“endogenous”) spatial and temporal cues are used to predict where (Space) and/or when (Time) targets will appear. The spatial location or temporal interval can be correctly (“validly”) or incorrectly (“invalidly”) cued. Adults are able to use both the valid Space and Time cues to respond quickly to the appearance of the targets when compared with a neutral cue condition [32]. When invalidly cued however, response times are much longer as the participant has to disengage attention from the invalidly cued location/time and shift it to where/when the target actually appears. It is important to note that space and time possess different characteristics that need to be taken into account when examining how attention moves within these dimensions. Space is experienced as a multidirectional dimension, in that attention can be oriented to any point in space. Being incorrectly or “invalidly” cued to either the right or the left is disruptive to the orienting of attention in space. By contrast, time is experienced as a unidirectional dimension, with time, or at least our everyday concept of time, always flowing forwards. Because of the unidirectionality of “time’s arrow”, the elapse of time itself carries temporally predictive information. If an expected event has not yet occurred, the expectation that it will occur at the next possible moment increases as the delay lengthens: just imagine yourself waiting for a late-running bus. The phenomenon underlying this subjective experience is known as the Hazard Function, which is defined formally as the increasing conditional probability over time that an event will occur given that it has not already occurred [36]. In the laboratory, this translates empirically into faster responses for events occurring after longer delays or “foreperiods” [37], and is known in the literature as the Variable Foreperiod (FP) Effect [38]. In terms of the attentional orienting task, this means that being invalidly cued to a short interval (i.e. the target is unexpectedly delayed) does not have the same behavioural cost as being invalidly cued to the long interval (i.e. the target is unexpectedly premature).

Although voluntary orienting of attention in time has not yet been investigated in children, there is evidence that children implicitly make use of the hazard function to predict the time of target onset. Children aged from 6–7 years onwards show the variable FP effect (i.e. RTs are faster for targets appearing after a long interval (or FP) than a short one) and the strength of this effect grows linearly as a function of age [5]. Moreover, children from 6–13 years old also show sequential effects [5, 39]: in other words, RT is modified by the duration of the FP on the previous trial [37, 40, 41]. Sequential effects index an automatic or unintentional form of temporal attention [42–44] but are asymmetrical: the length of the previous trial’s FP influences RT for trials with short FPs, but not for trials with long FPs. Specifically, RTs are slower for short FP trials than long FP trials only if the previous trial’s FP was longer. Although both variable FP and sequential effects index the implicit use of temporal information, sequential effects reflect a more automatic attentional process than variable FP effects. For example, sequential effects are resistant to dual-task interference whereas variable FP effects are attenuated [43]. Indeed, this functional distinction is reflected in the different developmental trajectories of variable FP and sequential effects: Vallessi and Shallice (2007) demonstrated that sequential effects were present earlier in development (4–5 years old) than variable FP effects (from 6 years old), although the characteristic asymmetry of the sequential effect began to be seen only by age 6 [5, 43]. Overall, these data suggest that children attend to the implicit temporal characteristics of a task in order to optimize their performance.

The primary aim of this study was to determine whether children were able to voluntarily orient their attention in time, as well as in space. We also aimed to confirm earlier findings (e.g.[5]) that children implicitly orient their attention in time, as indexed by the variable FP effect and sequential effects. At the other end of the developmental spectrum, it has been demonstrated that the orienting of attention in both space [45] and time [46] deteriorates in older adults. Zanto and colleagues used a time-only cueing design with 20 young adults and 21 elderly participants. The elderly adults were unable to use temporally informative cues to improve response times, but they did make use of the hazard function, in that response times were faster for targets appearing after a long interval than after a short one. The authors concluded that aging is associated with a deficit in orienting attention over time, due to deterioration in the brain mechanisms associated with temporal expectation [46]. Successful use of the hazard function however, suggests that even though elderly adults could not direct their attention in time voluntarily, they could direct attention implicitly. The question we now ask is whether, in children, these two forms of temporal expectation are also developmentally dissociable?

## Method

### Participants

Thirty typically developing children (11 females) and 32 adults (24 females) took part in the study. The mean age of the children was 11.0 years (SD 2.2, range 6–16) and the mean age of the adults was 26.9 years (SD 7.5, range 18–43). Twenty-five of the children were from primary schools in Belfast, Northern Ireland and 5 were from primary schools in regional Victoria, Australia. The adults were students and colleagues from the School of Psychological Sciences at the University of Melbourne.

Ethics approval was obtained from the Office for Research Ethics Committees Northern Ireland, the School of Psychology Ethics Committee, Queen’s University Belfast, the University of Melbourne Human Research Ethics Committee and the Catholic Education Offices in the Archdioceses of Melbourne and Sandhurst, in accordance with the 1964 Declaration of Helsinki. After complete description of the study to the participants, written informed consent was obtained from parents of all the children and from all of the adult participants.

### Experimental task

**Fig 1:**
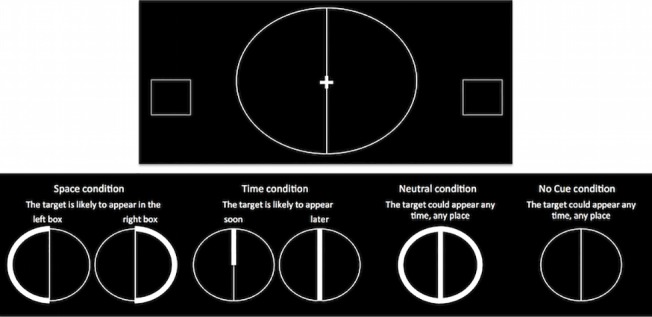
The stimulus display of the spatial and temporal orienting task. The figure above shows the central cue stimulus and peripheral target boxes. The figure below shows how the central cue stimulus changed appearance according to each of the four tasks.

All participants performed a modified version of the spatial and temporal orienting task [32], presented on a computer using E-Prime. The participants were presented with a background visual display of a central stimulus and two peripheral boxes (see Fig 1). They were asked to focus their gaze on the central stimulus and to use their peripheral vision to detect a target (either a + or x figure) that would appear in one of the two boxes. The central stimulus changed its appearance to present the participant with information about where (Space Cue) or when (Time Cue) the target would appear (see Fig 1). The participant was asked to use this information to respond to detection of the target as quickly as possible, by pressing the left side of an external mouse. The participants did not have to discriminate the target but simply to detect it. The central stimulus was based on the shape of a clock-face, to simplify the cue for children and to emphasise the concept of ‘time’.

There were four Cue conditions. In the Space Cue condition, either the left or right side of the clock face would brighten, indicating that the target would most likely appear in either the left or right box, respectively. In the Time Cue condition, either the short or long inner line, the “hand of the clock”, would brighten, indicating that the target would most likely appear either sooner (short line, 500ms) or later (long line, 1100ms). In the Neutral Cue condition, the entire central stimulus brightened, revealing no specific information about the appearance of the target, but which was relatively arousing. In the No Cue condition, the central stimulus did not change, revealing no information, nor providing any exogenous arousing properties.

A trial started with presentation of the background visual display of the central stimulus and two peripheral boxes, for a period of 600, 700, 800, 900 or 1000ms (i.e. the inter-trial interval), which was randomized across trials. The Cue stimulus then appeared for 100ms. The background visual display was then presented for one of the two stimulus-onset-asynchrony (SOA) intervals (500ms or 1100ms), after which the target appeared in one of the two boxes for 100ms. The trial ended with presentation of the background visual display for 1500ms, during which time the participants gave their speeded response.

The four Cue conditions were presented in separate blocks, in a randomized order. For the Space and Time Cue conditions, 32 valid and 8 invalid trials (40 trials) were presented in each of three blocks (120 trials altogether). In a valid trial, the target appeared where (Space) or when (Time) the central stimulus had indicated it would appear. In an invalid trial, the target appeared at the alternative spatial location or moment in time. For the Neutral and No Cue conditions, 16 trials were presented in each of three blocks (48 trials altogether).

Each block lasted approximately 2 to 3 minutes and participants were able to take breaks between the blocks. Participants were provided with a set of 32 training trials for each condition, to ensure they understood task instructions and, importantly, to learn the temporal association between cue-type and SOA in the Time condition. The training blocks contained wholly valid trials. After training, the participants were asked to identify the meaning of each of the cues to ensure understanding of the cues and were reminded to detect the targets as quickly as possible.

### Procedure

The child participants were tested in a quiet room at the child’s school and the adult participants were tested in a quiet testing room in the School of Psychological Sciences at the University of Melbourne.

### Data analysis

RTs of less than 100ms (errors of omission and extremely fast responses) were excluded from analyses. For each participant, the mean response time (RT) in ms was calculated separately for the valid and invalid trials at the 500 and 1100ms SOAs for the Space and Time Cue conditions, and at the two SOAs for the Neutral and No Cue conditions. Group means (M) and standard deviations (SD) for each trial type were calculated. The data were normally distributed, with no outliers.

The raw response time data from the experiment are available in S1 Dataset.

### Statistics

To determine the *validity effect*, a four-way mixed factorial ANOVA involving Group (Child, Adult), Cue Type (Space, Time), Validity (Valid, Invalid) and SOA (500ms, 1100ms) was analysed. To determine the *FP* and *sequential effects*, a three-way mixed factorial ANOVA involving Group (Child, Adult), SOA of the current trial i.e. SOA(n) (500ms, 1100ms) and SOA of the previous trial i.e. SOA(n-1) (500ms, 1100ms) was analysed with the neutral trials only. To determine the *alerting effect*, a three-way mixed factorial ANOVA involving Group (Child, Adult), Cue Type (Neutral, No Cue) and SOA (500ms, 1100ms) was analysed. Spearman’s correlation coefficient was used to assess the relationships between Age and the Validity effect (i.e. invalid-valid RTs) for the Space and Time cues at 500ms SOA (to avoid interference from the variable FP effect), the variable FP effect for the Neutral cue, and the Sequential effect for the Neutral cue. We examined both groups taken as a whole (age 6–43) as well as each group individually (child/adult). The alpha level was set to 0.05 and Bonferroni-adjustments were made for pair-wise comparisons.

## Results

### Spatial and temporal validity effects

**Fig 2:**
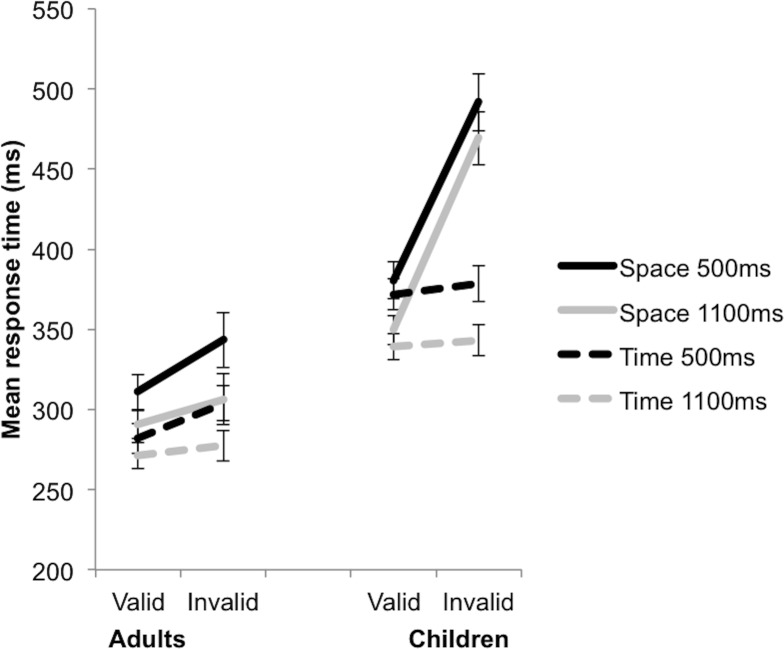
A significant Group by Validity by Cue interaction. This indicates the presence of both spatial and temporal validity effects in the adults, but only a spatial, not a temporal, validity effect in the children. In addition, a significant Group by Cue by SOA interaction revealed that RTs were faster at long SOAs (1100ms) than at short SOAs (500ms) in both groups, though in adults this effect was less pronounced in the temporal condition. Error bars reflect standard errors.

Group [F(1,60) = 45.490, p <. 001, η_p_^2^ =. 431], Validity [F(1,60) = 67.239, p <. 001, η_p_^2^ =. 528] and Cue [F(1,60) = 64.273, p <. 001, η_p_^2^ =. 517] main effects were further explained by a significant Group by Validity by Cue interaction, F(1,60) = 35.311, p <. 001, η_p_^2^ =. 370 (see Fig 2). This interaction was broken down by Group. For the Adults, there were significant Cue and Validity main effects. Participants responded to Valid trials significantly faster than to Invalid trials (see Table 1), (p <. 001). They also responded to the Time cue trials significantly faster than to the Space cue trials, (p <. 001). There was no significant Cue by Validity interaction F(1,31) = 3.061, p =. 090, η_p_^2^ =. 090. By contrast, for the Children, there was a significant Cue by Validity interaction, F(1,29) = 45.709, p <. 001, η_p_^2^ =. 612. The children showed a validity effect for the Space but not for the Time cue (Fig 2). For the Space cue, the RT for the Valid trials was significantly faster than for the Invalid trials, p <. 001. For the Time cue, however, there was no significant difference in RT between the Valid and Invalid trials, p =. 288. The three-way interaction was therefore driven by the significant temporal validity effect in adults and the lack of a significant temporal validity effect in children.

**Table 1:** Mean and standard deviation (in parentheses) measures of response time, in milliseconds, for the Adult and Child groups on the various levels of the Cue (Space, Time), Validity (Valid, Invalid), and Stimulus Onset Asynchrony (SOA) (500ms, 1100ms) independent variables of the spatial and temporal orienting task.

	Space Valid 500ms	Space Invalid 500ms	Validity effect	Space Valid 1100ms	Space Invalid 1100ms	Validity effect	Time Valid 500ms	Time Invalid 500ms	Validity effect	Time Valid 1100ms	Time Invalid 1100ms	Validity effect
Adults	311 (42)	344 (59)	33	291 (36)	306 (47)	15	282 (36)	304 (42)	22	271 (31)	277 (34)	6
Children	381 (79)	492 (126)	111	350 (61)	469 (121)	119	372 (67)	379 (77)	7	339 (57)	343 (69)	4

The difference between the Invalid and Valid trials per condition is noted as the Validity effect. The small temporal validity effects at 1100ms were expected, and are due to the influence of the variable FP effect on temporal orienting.

A significant SOA main effect, F(1,60) = 68.121, p <. 001, η_p_^2^ =. 532, was further explained by a significant Group by Cue by SOA interaction, F(1,60) = 4.776, p =. 033, η_p_^2^ =. 074. This interaction was broken down by Group. For the Adult group, significant SOA [F(1,31) = 37.211, p <. 001, η_p_^2^ =. 546] and Cue [F(1,31) = 44.544, p <. 001, η_p_^2^ =. 590] main effects were further explained by a significant Cue by SOA interaction, F(1,31) = 5.544, p =. 025, η_p_^2^ =. 152. The RTs following both the Space (p <0.001) and Time (p <0.001) cues were significantly faster at 1100ms compared with the 500ms SOA, indicative of the variable FP effect, but this effect was smaller following the Time cue (Fig 2). For the Child group, there was also a significant main effect of SOA, F(1,29) = 32.237, p <. 001, η_p_^2^ =. 526, with faster RTs at the 1100ms than 500ms SOA, but no significant Cue by SOA interaction, F(1,29) = 1.143, p =. 294, η_p_^2^ =. 038.

### Variable Foreperiod and Sequential effects

**Fig 3:**
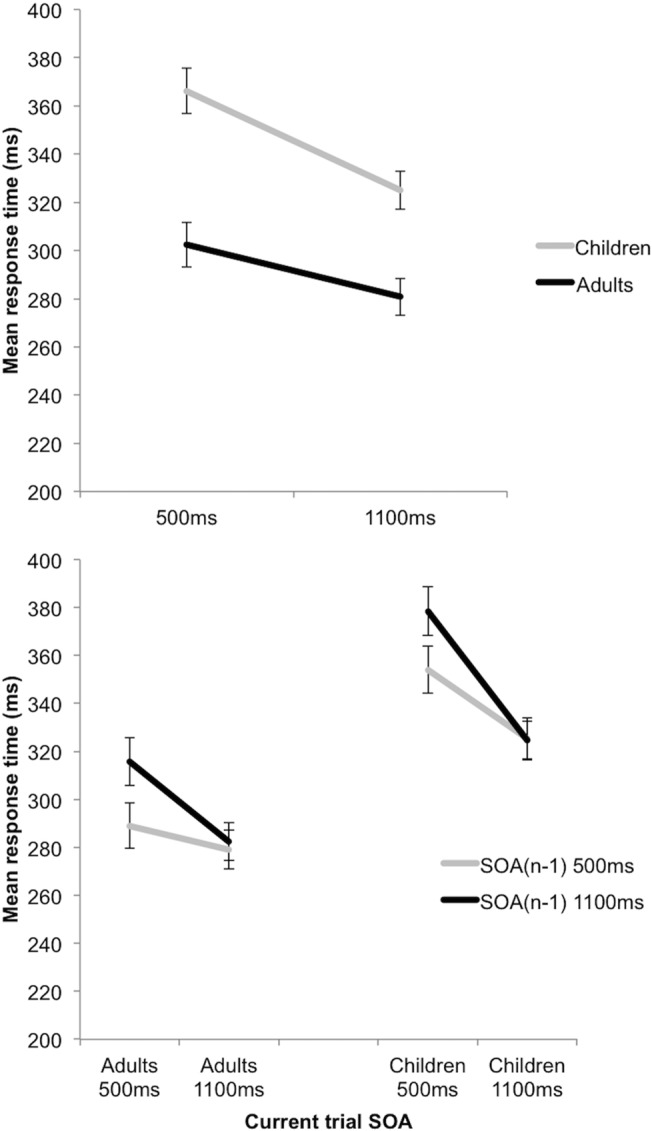
Variable foreperiod and sequential effects. Above: A significant interaction between Group and SOA(n) in Neutral trials. This indicates the presence of variable foreperiod (FP) effects in both groups, though with a greater effect demonstrated by the children. The variable FP effect reflects faster response times for events occurring after long, rather than short, delays (or “foreperiods”). Error bars reflect standard errors. Below: A significant SOA(n) x SOA(n-1) interaction in Neutral trials. This indicates the presence of sequential effects, in both groups equally. The sequential effect describes the phenomenon that RTs are faster for long FP trials than short FP trials only if the previous trial’s FP was long. The asymmetric nature of the sequential effects (the length of the previous (n-1) SOA influences RT only when the SOA of the current (n) trial is short) can be seen for both groups. Error bars reflect standard errors.

Significant Group and SOA(n) main effects were further explained by a significant Group x SOA(n) interaction, F(1,60) = 8.189, p =. 006, η_p_^2^ =. 120 (see Fig 3 and Table 2). Both the adult (p <. 001) and child (p <. 001) Groups showed the variable FP effect, with faster RTs at 1100ms SOA compared with 500ms, but with a greater difference demonstrated by the children. The adults were significantly faster than the children, at both the 500 (p <. 001) and 1100ms (p <. 001) SOA, but with a smaller difference found at 1100ms.

**Table 2:** Mean and standard deviation (in parentheses) measures of response time, in milliseconds, for the Adult and Child groups on the various levels of the Cue (Neutral, No Cue), and Stimulus Onset Asynchrony (SOA) (500ms, 1100ms) of the spatial and temporal orienting task.

	Neutral 500ms	Neutral 1100ms	No Cue 500ms	No Cue 1100ms
**Adults**	304 (36)	281 (32)	311 (36)	289 (38)
**Children**	366 (64)	325 (54)	371 (64)	339 (68)

A main effect of SOA(n-1) was further explained by a significant SOA(n) x SOA(n-1) interaction, F(1,60) = 20.627, p <. 001, η_p_^2^ =. 256 (see Fig 3 and Table 3). RTs were significantly slower when a current Short SOA(n) trial was preceded by a Long SOA(n-1) trial than by a Short SOA(n-1) trial (p <. 001) i.e. the Sequential effect. By contrast, RT was not significantly different whether a current Long SOA trial was preceded by a Short SOA(n-1) trial or a Long SOA(n-1) one (p =. 691), demonstrating the asymmetric nature of the Sequential effect. This SOA(n-1) by SOA interaction was therefore driven by a significant influence of the preceding trial’s SOA for Short SOA(n) but no significant effect of the preceding trial’s SOA for Long SOA(n).

**Table 3:** Mean and standard deviation (in parentheses) measures of response time, in milliseconds, for the Adult and Child groups on the various levels of the previous trial SOA(n-1) (500ms,1100ms) and the current trial SOA(n) (500ms, 1100ms) of the spatial and temporal orienting task.

	SOA(n-1) 500ms	SOA(n-1) 1100ms	SOA(n-1) 500ms	SOA(n-1) 1100ms
	SOA(n) 500ms	SOA(n) 500ms	SOA(n) 1100ms	SOA(n) 1100ms
**Adults**	289 (40)	316 (40)	279 (31)	282 (36)
**Children**	354 (65)	379 (69)	325 (58)	325 (52)

There were no two- or three-way interactions between Group and SOA(n-1), suggesting that both children and adults were demonstrating Sequential effects equally.

### Alerting effects

**Fig 4:**
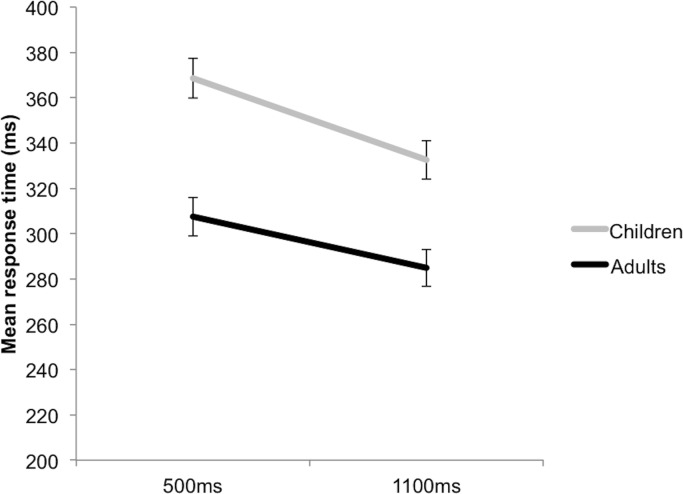
A significant Group x SOA interaction across Neutral and No Cue trials. This again demonstrates the variable foreperiod (FP) effect in both groups, though the effect was stronger in the children. Error bars reflect standard errors.

There was a significant main effect of Cue, with all participants responding significantly more quickly to the Neutral compared to the No Cue, F(1,60) = 4.386, p =. 040, η_p_^2^ =. 068. There was no significant Group x Cue interaction, F(1,60) =. 055, p =. 816, η_p_^2^ =. 001, suggesting that both groups were equally alerted by the Neutral cue. Significant Group and SOA main effects were further explained by a significant Group by SOA interaction, F(1,60) = 6.746, p <. 012, η_p_^2^ =. 101 (see Fig 4). Across Neutral and No Cue conditions, both the adult (p <. 001) and child (p <. 001) groups responded significantly more quickly at the Long SOA compared to the Short SOA, though this Variable FP effect was stronger for the children. The adults were significantly faster than the children in responding both at the Short (p <. 001) and Long (p <. 001) SOAs.

### Correlational analyses

Correlations were used to investigate potential relationships between Age and the spatial or temporal Validity effect. There was a significant negative correlation between Age and the spatial Validity effect, *r*_*s*_ = -.458, 95% BCa CI [-.630,-.243], p <. 001, and a significant positive correlation between Age and the temporal Validity effect, *r*_*s*_ =. 263, 95% BCa CI [.014,. 487], p =. 039. When these correlations were conducted for each group (Adults/Children) separately however, these significant effects disappeared, Adult Space *r*_*s*_ =. 126, 95% BCa CI [-.237,. 453], p =. 493; Adult Time *r*_*s*_ = -.054, 95% BCa CI [-.379,. 254], p =. 768, Child Space *r*_*s*_ = -.224, 95% BCa CI [-.568,. 095], p =. 234, Child Time *r*_*s*_ = -.014 95% BCa CI [-.416,. 454], p =. 943. This suggests that the significant correlations found for the entire group most probably reflect categorical between-group differences rather than parametric age-dependent relationships. An alternative, though not mutually exclusive, explanation for the lack of significant correlation in the children is an insufficient age-range: the majority of the children (n = 27) were aged between 10–13, with only 6 children aged less than 10 and just one aged greater than 13.

There were no significant correlations between Age and the Foreperiod or Sequential effects.

## Discussion

There were four main findings from this study. First, children aged 6–16 (mean age 11 years) did not show a significant slowing in mean RT in response to the invalid Time cue compared with the valid Time cue, suggesting that they were not using the Time cues to voluntarily direct their attention in time. In contrast, the adult group did make use of the valid Time cues to speed their responses. Second, children, like adults, were able to make use of the hazard function and showed the variable FP effect, in that they made significantly faster responses to targets appearing at the long (1100ms) SOA compared with the short (500ms) SOA. Third, children, like adults, showed the asymmetric sequential effect, in that their responses were significantly slower for short SOA trials when the previous trial had a long SOA, but RTs for long SOA trials were uninfluenced by the previous trial’s SOA. Fourth, children, like adults, responded more slowly to spatially invalid cues than valid cues, suggesting both groups used the spatial cue to speed responding. These findings suggest first and foremost that children in this age range cannot use symbolic temporal cues to voluntarily direct their attention in time. In other words, the ability to voluntarily use Time cues to speed one’s RT is a skill that develops after early adolescence. This cross-sectional developmental study therefore suggests that the skill of using endogenous temporal cues to voluntarily orient attention through time is on a different developmental trajectory compared with the skill of using spatial cues. Our findings further show that even though these children cannot voluntarily direct attention in time, they can nevertheless make use of the implicit temporal characteristics of stimulus presentation (as shown by variable FP effects and sequential effects) to direct attention in time in a more automatic or involuntary manner.

The fact that children showed both variable foreperiod and sequential effects supports the findings of previous research investigating these effects in children [5, 39]. Moreover, the contrast between their successful use of implicit temporal information to direct attention, but unsuccessful use of endogenous temporal cues to voluntarily direct attention is reminiscent of the dissociation reported by Zanto and colleagues with older adults (mean age of 70 years old) [46]. The use of cues to voluntarily direct attention through time may be a cognitive skill that develops from early adolescence and then diminishes in older adulthood.

Our data also have important cognitive implications by providing developmental evidence of dissociation between implicit and voluntary forms of temporal attention and thereby neatly complementing prior behavioural, electrophysiological and neuropsychological findings. For example, Rohenkohl et al (2011) and Breska and Deouell (2014) have shown that temporal predictability induced either implicitly by regular rhythms or voluntarily by endogenous cues independently speeded RTs in a target detection task, but their effects did not combine (interact) to improve performance to an even greater extent [47, 48]. Similarly, an electrophysiological marker of temporal preparation, the Contingent Negative Variation (CNV), has been shown to be modulated both by implicit (and automatic) sequential effects and by voluntary endogenous cues, though these effects were independent and additive, rather than mutually interactive [49]. In addition, electrophysiological [50] and neuroimaging [4, 51] evidence reveals that the neuroanatomical signatures of implicit and voluntary forms of temporal attention are quite distinct: implicit temporal attentional processes indexed by the variable FP effect recruit right prefrontal areas whereas voluntary temporal attention induced by endogenous cues recruits left-lateralised premotor and parietal areas.

It is important however to distinguish between implicit forms of temporal attention that are induced by the hazard function (and indexed by variable FP effects) from those that are induced by rhythmic stimulus presentation. Trivino et al (2011) found that patients with right prefrontal lesions were unable to benefit from endogenous temporal cues or from the hazard function [52]. Patients however were unimpaired in their ability to benefit from temporally predictable rhythms. Our own findings in children indicate that implicit forms of temporal attention indexed by variable FP and sequential effects develop earlier than more voluntary forms of temporal attention. Trivino et al’s findings in patients suggest that rhythm-induced temporal expectation may develop even earlier, a finding supported by electrophysiological evidence that infants as young as 10 months old can detect a temporal deviant in a sequence of rhythmically (isochronously) presented stimuli [53].

One obvious question that remains to be answered is why can’t the children in our sample use the Time cue to voluntarily orient their attention through time? One possibility is that the Time cues are conceptually more demanding than the Space cues, and even though the Time cues were simplified in this study compared with the cues from the original spatial and temporal orienting task [32], the children did not appear to use them to aid attentional orienting.

Alternatively, the spatial uncertainty of target appearance may have diminished the utility of the Time cue for the children. In an electrophysiological investigation of cued target detection in adult volunteers, Doherty et al (2005) found that the amplitude of the sensory P1 component of the event-related potential was enhanced by spatial predictability but not by temporal predictability [54]. If temporally predictable targets were also spatially predictable however, then the sensory P1 component was enhanced to an even greater extent than spatial predictability alone. Rohenkohl et al (2014) have recently confirmed these findings behaviourally, showing that perceptual discrimination was improved by endogenous temporal cues only if target location was also known in advance [55]. In other words, temporal predictability is most effective when combined with spatial predictability. Indeed, preliminary evidence from our lab shows that children can use a Time cue to speed responding when the spatial location of the target is known in advance.

A final, more neural, possibility for why children did not benefit from the Time cues relates to the differential developmental maturation of discrete brain areas throughout childhood. An obvious explanation is that the brain regions necessary for voluntary orienting of attention in time develop later than the regions necessary for automatically or implicitly orienting in time. Functional neuroimaging investigations in adults however suggest that despite being functionally distinct, voluntary and automatic forms of temporal attention are actually neuroanatomically rather similar, recruiting the same regions of the brain. Endogenous temporal cues consistently activate left inferior parietal cortex, as compared either directly to spatial orienting [32] or to neutral cue conditions [56, 57], as do more automatic forms of orienting induced by visual trajectories [58] or visual [59] and auditory rhythms [60]. It is possible, however, that different regions of the brain would be engaged by voluntary temporal orienting in the developing childhood brain as compared to the mature adult brain. Indeed, an example from the spatial orienting literature indicates that this is a plausible explanation: using MEG, Bayless and colleagues demonstrated that younger participants (7–8 years) performed a spatial orienting task with slower and more diffuse neural activation compared with the 12–13 year old group, who performed the task with a similar pattern of activation as adults [61]. It may only be through experience and interaction with the dynamic world that the neurofunctional circuits underlying voluntary and automatic forms of attention converge in adulthood.

The endogenous temporal and spatial cues used here were intended to induce voluntary orienting. Spatial arrow cues, however, similar to those used here, are thought to elicit automatic, as well as voluntary, orienting [62–64]. Given our evidence that children orient their attention in time automatically, but not voluntarily, it is possible that children were also using our spatial cues to orient attention in space automatically, rather than voluntarily. In support of this, Ristic and Kingstone (2009) have found that preschool children extracted spatial information from arrow cues both voluntarily *and* automatically, in an additive manner [65]. Indeed, if the children in our own experiment were using spatial cues to orient attention automatically as well as voluntarily, this may explain the exceptionally large spatial validity effects we found in children compared to adults. Adults, however, also orient attention automatically and voluntarily to predictive arrow cues in a super-additive manner [64]. An alternate explanation is that in the spatial condition the children may orient automatically to a greater degree than adults and then experience more difficulties with disengaging attention to reorient. It would therefore be advisable to repeat our experiment using spatial cues that engage only voluntary attention. Olk (2014) has recently shown that when both temporal and spatial cues are designed to induce voluntary orienting only (i.e. thick/thin lines indicate right/left or long/short), validity effects in adults are more comparable across the spatial and temporal dimensions [66]. We plan to use this better-matched version of the spatial and temporal orienting task in children to determine whether the distinct developmental trajectories we have found are due to differences in automatic/voluntary forms of orienting, or whether they truly reflect differences in processing of spatial versus temporal information.

In conclusion, the children in this study were able to make use of the Space cues to speed their responses to targets (using voluntary and/or automatic mechanisms), yet were unable to make use of the Time cues in the same way, suggesting distinct developmental trajectories for spatial and temporal orienting of attention and/or differences in the ability to use different types of cues. Nevertheless, children were able to implicitly use the temporally predictive information inherent in the passing of time to speed responses. The dissociation in the RT benefits of explicit versus implicit temporal information suggests that automatic orienting of attention in time develops earlier than the more voluntary orienting required by endogenous temporal cues.

## Supporting Information

S1 Dataset The raw response time data for each participant. (XLSX)
